# A semantic union model for open domain Chinese knowledge base question answering

**DOI:** 10.1038/s41598-023-39252-w

**Published:** 2023-07-24

**Authors:** Huibin Hao, Xiang-e Sun, Jian Wei

**Affiliations:** School of Electronic Information, Yangtze University, Jingzhou, 434100 China

**Keywords:** Computer science, Scientific data, Information technology

## Abstract

In Open-domain Chinese Knowledge Base Question Answering (ODCKBQA), most common simple questions can be answered by a single relational fact in the knowledge base (KB). The abbreviations, aliases, and nesting of entities in Chinese question sentences, and the gap between them and the structured semantics in the knowledge base, make it difficult for the system to accurately return answers. This study proposes a semantic union model (SUM), which concatenates candidate entities and candidate relationships, using a contrastive learning algorithm to learn the semantic vector representation of question and candidate entity-relation pairs, and perform cosine similarity calculations to simultaneously complete entity disambiguation and relation matching tasks. It can provide information for entity disambiguation through the relationships between entities, avoid error propagation, and improve the system performance. The experimental results show that the system achieves a good average F1 of 85.94% on the dataset provided by the NLPCC-ICCPOL 2016 KBQA task.

## Introduction

The recent rapid development of large-scale knowledge bases (KBs) has significantly made open-domain KB question answering become a research hotspot in the field of natural language processing (NLP), which offers accurate answers to natural language (NL) questions. Thus, we considered the most common questions^1–3^ that contain an entity mentioned and link to an entity in a KB, but there may be multiple ambiguous entities with the same name. For example, the question “Who is the author of Journey to the West” contains the entity mentioned “Journey to the West” and can be answered with a fact triple (Journey to the West (novel), author, Wu Chengen), rather than other entities such as Journey to the West (movies).

Although significant progress has been made regarding the KB questions in the English answering system recently, the corresponding method is unsatisfactory for realizing the Open-domain Chinese knowledge base question answering (ODCKBQA), based on the following challenges:

(1) The first is to accurately find the entities in the KB, corresponding to the entity mentioned in the question, which is the process of entity disambiguation. Although too many entities with the same name are found in Chinese, fewer descriptions of entities are found in the questions. When abbreviations and aliases appear in entities, it becomes difficult to find the correct corresponding entities in the KB.

(2) To accurately match questions with structured semantics relation in the KB, the Chinese language comprises a rich language expression, which makes computers face challenges in accurately understanding the semantics of NL questions, especially in relational matching tasks.

To solve these two challenging issues, most previous methods divided the entity disambiguating and relation-matching tasks in ODCKBQA into two independent subtasks. However, these methods failed to consider the correlation between the subtasks and the problem associated with the error transmission. If the candidate entities and the connected relations are known, we can focus on the candidate entities closely related to the relationship to learn the semantic similarity between the question and the candidate entities in disambiguation. Thus, relational information is meaningful for entity disambiguation. This study proposes a Semantic Union Model (SUM), which takes full account of the impact of entity ambiguity, regards entity disambiguation and relation matching as complementary and highly related joint tasks, and uses the CoSENT^4^ model based on contrastive learning to draw similar sentence pairs closer and dissimilar sentence pairs far away in the vector space, so as to obtain a more differentiated semantic vector representation of questions and candidate entity-relation pairs.

We performed the experiments on the NLPCC-ICCPOL 2016 KBQA task to verify the suitability of the proposed SUM for the ODCKBQA application. Experimental results show that the method achieves the good performance when applied to simple open-domain questions in Chinese.

The main contributions of this paper are as follows: (1) A new SUM is proposed, which fully considers the impact of ambiguities between entities with the same name, and deep joint modeling of entity disambiguation and relationship matching tasks to avoid error transmission; (2) Using CoSENT model based on contrastive learning to learn questions and candidate entity relationship pairs to obtain more discriminative semantic vector representations; (3) Experiments on the NLPCC ICCPOL 2016 KBQA public evaluation dataset show that this method can achieve superior performance and verify the effectiveness of the method.

## Related work

In NLP, open-domain knowledge base question answering has been the focus of many researchers^2,3,5–8,17–20^ in the last few years. Most current state-of-the-art KBQA research methods employ semantic parsing-based (SP) and information retrieval-based (IR) methods.

In SP methods, the goal is to convert NL questions into equivalent logical expressions according to a specific grammar, complete the query of the KB, and obtain the answers^9–11^. Since the open-domain Chinese KB contains hundreds of thousands of relations, the SP methods face the problem related to the unregistered relation words. In these methods, the training set may face difficulty in covering such a large number, making it limited in ODCKBQA.

The IR methods first accurately locate the entities in the question, then maps the entities to the knowledge base, obtains all the connected relation and attribute value entities, and gets the answers by calculating the similarity between the question and them^2,12–20^. Bordes et al.^2^ proposed the vector embedding-based method to encode questions and answers, calculate the semantic similarity between the two, and sort them. Li et al.^12^ designed a multi-column convolutional neural network to capture the interactive information between questions and answers. Xie et al.^14^ apply Deep Structured Semantic Models (DSSM)^15^ based on convolutional neural network and bidirectional long short-term memory (BiLSTM)^16^ to calculate the similarity between the question and relationships. Lai et al^17^. used the word frequency and length features of entity to find entity mention in question and their corresponding entity in the KB, and then matched the corresponding relation based on word2vec word embedding cosine similarity and relational word attention methods. Later, a shallow method based on features and word embedding was proposed to generate candidate entities and relationships, and then deep CNNs were used to reorder these entity-relation pairs^18^.

With the development of pre-trained language models, some studies^6,19–21^ have used pre-trained language models to construct ODCKBQA. Liu et al.^6^ used a pre-trained language model BERT^22^ to learn the semantic representation of questions and candidate words. Li et al. ^19^ added the loss function in the entity mention recognition task and the relationship matching task to conduct joint modeling, trained the BERT model with shared parameters, and used the output of KB entities, text fuzzy matching and n-gram information to complete entity link, but they did not fully consider the ambiguity of entities with the same name but different semantics. Lin et al. ^20^ used unsupervised and fine-tuning methods to train the MT5 model to obtain the ability to convert answer sentences constructed through triples into question, and used the Roformer model to determine whether candidate sentences and question were similar or dissimilar. It uses fuzzy matching to search for candidate entities and their triples from KB based on the entity mention in the question. This method ignores ambiguous entities with the same name and does not consider the impact of ambiguous entities in the evaluation of the overall system.

These models mentioned above are unable to effectively consider and solve the impact of ambiguity between entities with the same name on ODCKBQA, and cannot accurately distinguish problems and candidate words with similar texts but significant semantic differences. Thus, this study proposes using the CoSENT model to learn more discriminative semantic vector representations of questions and candidate entity-relation pairs. Furthermore, we integrate entity disambiguation and relation-matching tasks into a unified SUM framework.

## Models and methods

**Figure 1:**
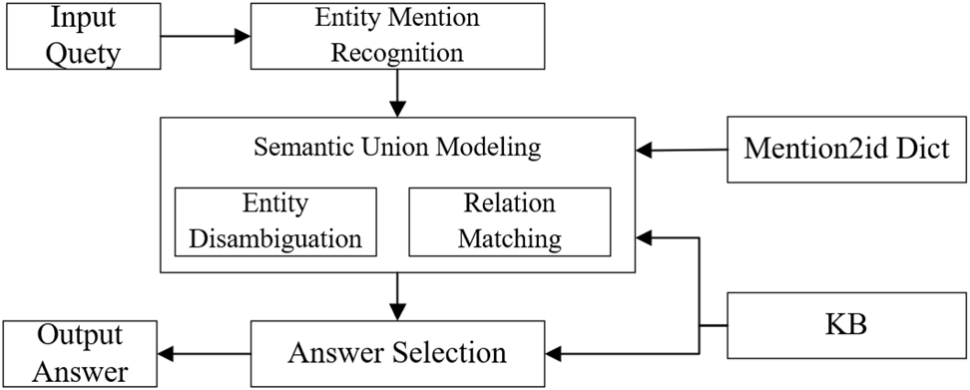
Overall ODCKBQA framework.

Figure 1 shows the overall ODCKBQA framework, comprising three subtasks: entity mention recognition, entity disambiguation, and relation matching. The mention2id dict provides candidate entities for entity disambiguation tasks. The entity reference recognition module identifies the subject entity reference that contains information from the input NL questions. However, entity mentions in NL interrogative sentences often represent multiple meanings, and entity disambiguation must find the exact corresponding entity in the KB. Moreover, the relations in question usually have different surface forms and are not easy to match the relations in the KB. The mismatch between NL questions and structured semantic knowledge base is a key challenge in ODCKBQA. We propose the CoSENT model to learn deeper semantic features and distinguish this semantic difference. Finally, the answer extraction module extracts answers from the KB using query statements through the entities and relationships obtained previously.

Traditional methods treat the two tasks of entity disambiguation and predicate matching as independent subtasks, ignoring their dependencies. Intuitively, candidate entities connected by similar predicates offer more information for entity disambiguation tasks and vice versa. When they act as independent tasks, error propagation will occur and subsequently affect the overall system performance. Thus, we propose a SUM that combines entity disambiguation and relation-matching tasks in a unified framework, considering a full account of the correlation between the two tasks.

### Base model

This section describes the BERT and CoSENT models used in this article.

#### BERT

**Figure 2:**
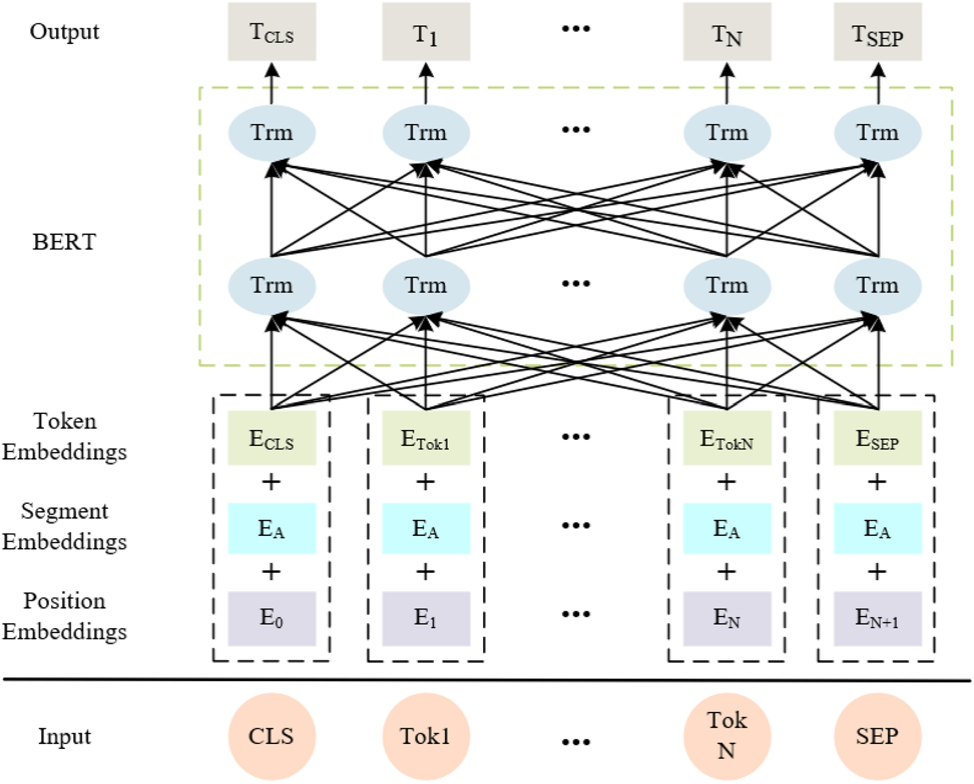
Structure of the BERT model.

Figure 2 shows the structure of the BERT model. The model input vector consists of three parts: Token Embeddings, Segment Embeddings, and Position Embeddings. Moreover, BERT adds a special [CLS] tag before the input sentence sequence, and the output vector corresponding to this tag is used as the semantic representation $\left\{ {q_{1} ,q_{2} , \cdots ,q_{N} } \right\}$ of the entire input sequence, usually used for classification tasks. Then, the model adds a special [SEP] tag after the sentence sequence token for sentence segmentation. We input the sequence representation of the question Q into the BERT model to get the vector representation of each word in the sentence:

1 $$H_{q} = BERT\left( Q \right)$$

where $H_{q} = \left\{ {h_{cls} ,h_{1} ,h_{2} , \cdots ,h_{N} ,h_{seq} } \right\}$, N is the length of the input sequence Q, and $h_{i}$ is the output vector representation of the BERT layer, corresponding to the $i$ th word.

##### CoSENT modele

The structure of the CoSENT model is similar to Sentence BERT^23^, uses two parameter-shared BERTs to form a Siamese neural network. The CoSENT model outputs respective semantic vectors of input sentences $U$ and $V$. Then, it pools them to derive fixed-size sentence embeddings and uses a cosine similarity function for similarity calculations and the cosine similarity formula as shown in Eq. (2):

2 $$similarity = cos\left( {U,V} \right) = \frac{U \cdot V}{{\left\| U \right\|\left\| V \right\|}} = \frac{{\sum\nolimits_{i = 1}^{n} {U_{i} \times V}_{i} }}{{\sqrt {\sum\nolimits_{i = 1}^{n} {\left( {U_{i} } \right)^{2} } } \times \sqrt {\sum\nolimits_{i = 1}^{n} {\left( {V_{i} } \right)^{2} } } }}$$

In the training phase of the CoSENT model, $h^{ + }$ is the set of all positive sample pairs, and $h^{ - }$ is the set of all negative sample pairs. For any positive sample pair $\left( {h_{i} ,h_{j} } \right) \in h^{ + }$ and negative sample pair $\left( {h_{k} ,h_{l} } \right) \in h^{ - }$, we develop the following:

3 $$\cos \left( {u_{i} ,u_{j} } \right) > \cos \left( {u_{k} ,u_{j} } \right)$$

where $u_{i} ,u_{j} ,u_{k} ,u_{j}$ is the sentence vector of $h_{i} ,h_{j} ,h_{k} ,h_{l}$ respectively. The loss function of the CoSENT model is shown in Eq. (4):

4 $$\log \left( {1 + \sum\limits_{{\left( {h_{i} ,h_{j} } \right) \in h^{ + } ,\left( {h_{k} ,h_{l} } \right) \in h^{ - } }} {e^{{\lambda \left( {\cos \left( {u_{i} ,u_{j} } \right) - \cos \left( {u_{k} ,u_{j} } \right)} \right)}} } } \right)$$

Among them, $\lambda$ is a hyperparameter greater than 0, taken as 15 in the subsequent experiments. The loss function is used to pull the representation of semantics of similar sentence pairs in the vector space and to move dissimilar sentence pairs in the retraining process to obtain a more discriminative sentence vector representation.

### Semantic union model

**Figure 3:**
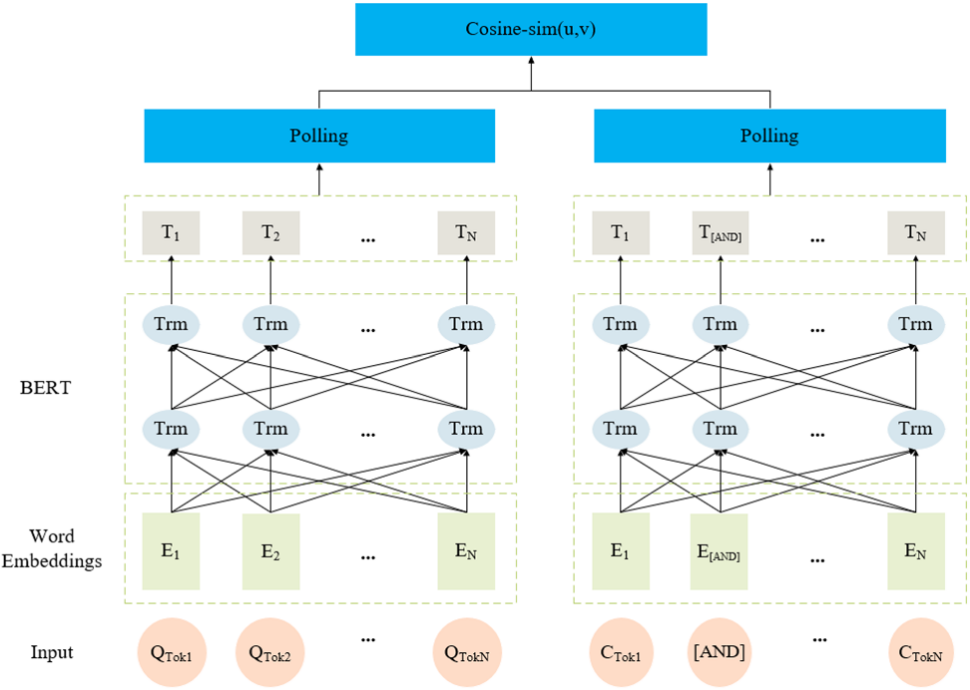
SUM framework.

Figure 3 shows the SUM framework, which uses the CoSENT to learn semantic vector representations of questions and entity-relation pairs to match entities and relations candidate fact triples, considering deeper semantic features. Note that question and entity-relation pairs use a BERT model with shared parameters to output semantic vectors.

First, we connect each candidate entity $e_{i}$ in the candidate entity set $E = \left\{ {e_{1} ,e_{2} , \cdots ,e_{l} } \right\}$ and its connected predicate set $R_{i} = \left\{ {r{}_{1}^{i} ,r{}_{2}^{i} , \cdots ,r{}_{n}^{i} } \right\}$, through a special [AND] identifier to form the candidate entity-relation pairs set $C = \left\{ {e_{1} r{}_{1}^{1} , \cdots ,e_{1} r{}_{n}^{1} ,e_{2} r{}_{1}^{2} , \cdots ,e_{l} r{}_{n}^{l} } \right\}$. Second, question Q and the candidate entity-relation pair set $C$ are input into the BERT layer to obtain their vector representations. Then, these vectors are fed into pooling layers separately to obtain fixed-size sentence embeddings, expressed as the following:

5 $$H^{q} = polling\left( {Bert\left( Q \right)} \right) = \left\{ {h{}_{1}^{q} ,h{}_{2}^{q} , \cdots ,h{}_{n}^{q} } \right\}$$

6 $$H^{c} = polling\left( {Bert\left( C \right)} \right) = \left\{ {h{}_{1}^{c} ,h{}_{2}^{c} , \cdots ,h{}_{n}^{c} } \right\}$$

The pooling layer uses the average pooling strategy by default and the cosine similarity function to calculate their similarity:

7 $$sim\_s = cos\left( {H^{q} ,H^{c} } \right)$$

where $sim\_s$ is the set of similarity scores between the question and the candidate entity-relation pair, and our minimized objective loss function is the same as Eq. (4).

Intuitively, some candidate relations provide semantic information for entity disambiguation. If we know the relationship in the question, we can exclude some candidate entities through their semantic information. For example, the question "How many pages do a dream of Red Mansions have?" contains the relative word "number of pages" corresponding to the word "how many pages." For entity disambiguation, it is reasonable to focus on candidate entities connected with "pages," such as "Dream of Red Mansions (novel)" rather than "Dream of Red Mansions (movie)." Therefore, we constructed a SUM to perform entity disambiguation and relation matching.

### Entity mention recognition

We used the BIO standard strategy to represent each word in the question. The entity mention recognition task is to identify the subject entity mentioned. We constructed a BERT-BiLSTM-CRF model with question Q as the input sequence, which consisted of a BERT layer, BiLSTM layer, and CRF layer, where the BERT layer structure was the same as that shown in Fig. 1. We input the sequence representation $\left\{ {q_{1} ,q_{2} , \cdots ,q_{N} } \right\}$ of the question Q into the BERT-BiLSTM-CRF model to obtain the label probability distribution of each word in the sentence:

8 $$Y = BERT - B{\text{i}}LSTM - CRF\left( Q \right)$$

where Y is the label probability distribution predicted by the model. We chose the label with the highest probability as the label of the word. We took the fields labeled B and I as the entity mentioned output for the BIO standard strategy.

### Entity disambiguation and relation matching

Since the entity mentioned in the question corresponds to multiple entities that have different meanings in the KB, entity disambiguation operations map the entity mentions in the question with a known unambiguous entity in the KB. Given the question $Q = \left\{ {x_{1} ,x_{2} , \cdots ,x_{n} } \right\}$ and the candidate entity set $E = \left\{ {e_{1} ,e_{2} , \cdots ,e_{l} } \right\}$, we use the CoSENT model to calculate the similarity between them and rank them, as shown in Eq. (9):

9 $$P^{e} {\text{ = CoSENT}}\left( {Q,E} \right)$$

where $P^{e}$ is the semantic similarity score between the question and the candidate entity.

Most entities in the KB are connected with multiple relationships. The relation-matching task scores each candidate relation according to the semantic similarity between each candidate relation of the question and the entity to identify the relation word that best matches the semantics of the question. After the entity disambiguation task, we obtain all its connected relations from KB, according to entity mentions, which form a candidate relation set $R = \left\{ {r_{1} ,r_{2} , \cdots ,r_{n} } \right\}$, where n is the number of candidate relations. We used the CoSENT model to obtain the semantic similarity score between question Q and the candidate relation $r_{i}$, as shown in Eq. (10):

10 $$P^{r} {\text{ = CoSENT}}\left( {Q,R} \right)$$

where $P^{r}$ is the semantic similarity score between the question Q and the candidate relation set $R$.

The above process executes the entity disambiguation task and the relation matching task, leading to the error’s transmission. If the entity selected by the entity disambiguation model deviates from the question, the relation-matching model will fail to find the correct relationship, thereby unable to find the correct answer in the KB. Here, the information from the relation-matching stage cannot be used in the entity disambiguation process. For example, some candidate entities do not have the correct relationship, which may still be selected in the entity disambiguation task, eventually leading to wrong results. Thus, we proposed SUM to complete the joint task of entity disambiguation predicate matching and calculated the semantic similarity of candidate entity-relation pairs and questions.

We performed fuzzy matching in the Neo4j graph database through the entity mentioned in the question to obtain candidate entity-relationship pairs. Then, we used the mention2id dictionary to filter them, retaining only the candidate entities and their relationships corresponding to the dictionary entity mentions. We also formed a set $C$ of candidate entity-relationship pairs. With the SUM model, we calculated the semantic similarity between the question and the candidate entity-relation pair set. Then, we selected the top N candidate entity-relation pairs:

11 $$P^{er} = SUM\left( {Q,C} \right)$$

where $P^{{{\text{er}}}}$ is the semantic similarity score of $Q$ and set $C$. We selected the candidate entity-relation pair with the highest score and obtained the corresponding answer from the Neo4j graph data through the CQL query statement for the answer.

## Experiment

We described the KB, data sets, parameter settings, and evaluation indicators. Then, we present the experimental results and analysis.

### Experimental setup

#### Knowledge base introduction

We gathered our dataset from the NLPCC ICCPOL 2016 KBQA datasets, which contained a training set of 14,609 question–answer pairs and a test set of 9870 question–answer pairs. This dataset provides a KB and a mention2id entity ambiguity dictionary, in which the KB contains 6,502,738 entities, 587,875 relations, and 43,063,796 triples. Each line in the KB file stores a text file, comprising a triple (entity, relationship, entity), and the mention2id dictionary includes 7,623,034 entity–entity pairs. The content of the KB is shown in Table 1.

**Table 1:** NLPCC ICCPOL 2016 KBQA Example of the KB.

Entity	Relationship	Entity/Value
高等数学(2004年高等教育出版社图书)	别名	高等数学
高等数学(2004年高等教育出版社图书)	书名	高等数学
高等数学(2004年高等教育出版社图书)	作者	仇庆久
高等数学(2004年高等教育出版社图书)	ISBN	704,011,885
高等数学(2004年高等教育出版社图书)	出版社	高等教育出版社

#### Datasets

The experiments are based on the dataset collected from the NLPCC ICCPOL 2016 KBQA datasets, comprising entity mentions, relations, and answers to questions. For the entity mention recognition task, we labeled the entity mentions in the question using the BIO notation, based on the entity mentions provided by the original dataset. For the entity disambiguation task, we obtained the candidate entities according to the mention2id dictionary and the mention of the question. We also queried the corresponding entity in the KB through the answer and relationship of the question, marked it as a positive example, and marked other candidate entities as a negative example. For the relation-matching task, we fetched all the relations connected to the correct entity from the KB and labeled the correct relations as positive examples and other relations as negative examples. For the joint task of entity disambiguation relation matching, we performed the fuzzy matching in the Neo4j graph database based on mentions to obtain candidate entity-relation pairs, which were filtered using the mention2id dictionary. Afterward, the correct entity-relation pairs are marked as positive examples, and other entity-relationship pairs are marked as negative examples. Table 2 shows the final datasets of each subtask.

**Table 2:** Datasets of each subtask.

Task	Training set	Deving set	Testing set
Entity Mention Recognition	13,267	975	9870
Entity Disambiguation	60,522	6724	36,219
Relation Matching	132,388	14,709	102,589
Joint task of entity disambiguation relation matching	337,065	37,481	320,579

#### Parameters

We used the Chinese BERT base model to initialize the weights. For all models, we set the maximum sequence length to 64, the batch size to 32, and the epoch to 20. We minimized the loss function using Adam, and the learning rate was set to 2e-5. Then, we set the hyperparameter $\lambda$ of Eq. (4) to (15).

#### Evaluation metrics

We used $AverageF_{1}$ to evaluate the KBQA system performance. The formula $AverageF_{1}$ is defined as the following:

12 $$AverageF1 = \frac{1}{\left| Q \right|}\sum\limits_{i = 1}^{\left| Q \right|} {F_{i} }$$

where $F_{i}$ represents the F1 score for a question $Q_{i}$; $F_{i}$ is set to 0 if the generated answer set $C_{i}$ for $Q_{i}$ is empty or does not overlap the golden answers $A_{i}$ for $Q_{i}$. Otherwise, formulate $F_{i}$ as follows:

13 $$F_{i} = \frac{{2 \cdot \frac{{\# \left( {C_{i} ,A_{i} } \right)}}{{\left| {C_{i} } \right|}} \cdot \frac{{\# \left( {C_{i} ,A_{i} } \right)}}{{\left| {A_{i} } \right|}}}}{{\frac{{\# \left( {C_{i} ,A_{i} } \right)}}{{\left| {C_{i} } \right|}} + \frac{{\# \left( {C_{i} ,A_{i} } \right)}}{{\left| {A_{i} } \right|}}}}$$

where $\# \left( {C_{i} ,A_{i} } \right)$ represents the number of answers that appear in both $C_{i}$ and $A_{i}$; $\left| {C_{i} } \right|$ and $\left| {A_{i} } \right|$ denote the number of answers in $C_{i}$ and $A_{i}$, respectively.

$Accuracy@N$ represents the average accuracy of the candidate set with the topN scores containing the correct results.

### Experimental results and analysis

For the entity mention recognition module, we used the BERT-BILSTM-CRF model to identify entity mentions in question sentences. We achieved entity-level accuracy of 97.41% using the BERT-BILSTM-CRF model, and 98.05% after adding manual rules. The next step is to analyze the results of the following experiments:

(1) As revealed in Table 3, the CoSENT model in the entity disambiguation task is superior to other models, assisting in obtaining deeper semantic information. In the training stage, the CoSENT model optimizes the cos value of two sentences to obtain more differentiated semantic information. Compared with the CoSENT model, the BERT model and the Sentence-BERT model record a drop in performance by 0.73% and 3.04%, respectively, when using the classification model in the training phase. The ability of CoSENT model to extract Semantic information is better than Siamese BiLSTM and Siamese CNN models built using traditional neural networks.

**Table 3:** Different model accuracy of entity disambiguation (%).

Model	Accuracy@1	Accuracy@2	Accuracy@3
Siamese BiLSTM	87.85	92.58	94.59
Siamese CNN	88.04	92.68	94.88
BERT-Softmax	89.12	93.15	95.01
Sentence-BERT	86.81	91.98	94.19
CoSENT	89.85	93.43	95.31

(2) Table 4 presents the experimental results of the relation-matching task. Since entity mention in question may affect the effect of model learning, we conduct a set of experiments on whether entity mention in questions carries mask operation. The experimental results show that after masking the entity mention of the questions in the dataset, the effect of the model is improved, and the BERT-Softmax(mask) of the interactive model is slightly better than the CoSENT(mask) model of the representation model, with the best performance. In the representation model, CoSENT based on contrastive learning outperforms Siamese BiLSTM and Sentence BERT models, and proved the superiority of contrastive learning loss.

**Table 4:** Different model accuracy of relation matching (%).

Model	Accuracy@1	Accuracy@2	Accuracy@3
Siamese BiLSTM	91.32	95.67	97.22
Siamese BiLSTM(mask)	92.34	96.23	97.35
BERT-Softmax	94.46	97.63	98.61
BERT-Softmax(mask)	94.91	98.07	98.88
Sentence-BERT	91.15	95.57	97.03
Sentence-BERT(mask)	91.81	95.96	97.38
CoSENT	92.72	96.61	97.71
CoSENT(mask)	93.84	97.45	98.45

(3) As shown in Table 5, the experimental results of the Entity disambiguating relation matching joint task show that the mask operation has a certain effect on the entity mentioned in the question and candidate entity-relation pairs. The effect of the CoSENT model is 0.12% higher than that of the BERT model,2.07% higher than that of the Sentence-BERT model and1.94% higher than that of the Siamese BiLSTM. These results prove that the CoSENT model can learn deeper semantic information. We also performed an experiment on the prediction speed of the model. The experimental results show that the expression model CoSENT is much faster than the interactive model BERT in terms of prediction speed, making it more suitable for large-scale semantic matching tasks.

**Table 5:** Entity disambiguation relationship matches the accuracy (%) and speed (ms) of joint task.

Model	Accuracy@1	Rate
Siamese BiLSTM	84.26	45.42
Siamese BiLSTM(mask)	85.71	43.67
BERT-Softmax	87.29	843.18
BERT-Softmax(mask)	87.53	809.29
Sentence-BERT	84.35	118.61
Sentence-BERT(mask)	85.58	116.51
CoSENT	87.17	124.52
CoSENT(mask)	87.65	123.95

(4) We also performed the experiments on the NLPCC ICCPOL 2016 KBQA datasets, and the evaluation index used in the final results of the official evaluation was the average F1 value. The overall system uses the BERT-BILSTM-CRF model in the entity reference identification module and performs mask operations in the relation matching and joint task models. The final overall KBQA results are shown in Table 6. The experimental results show that an SUM model, which is an entity disambiguation relation matching task, has advantages over the pipeline in ODCKBQA.

**Table 6:** Overall KBQA results (%).

Model	Accuracy@1
Pipeline (Siamese BiLSTM)	79.53
Pipeline(BERT-Softmax)	83.39
Pipeline(Sentence-BERT)	78.15
Pipeline (CoSENT)	82.67
SUM (Siamese BiLSTM)	84.03
SUM(BERT-Softmax)	85.82
SUM(Sentence-BERT)	83.91
SUM(COSENT)	85.94

Table 7 compares all the results^6,14,17–20,24,25^, which participate in the NLPCC-ICCPOL 2016 KBQA evaluation task. The experimental results show that the average F1 score of our proposed SUM is 85.94%, which is superior to other pipeline models that using many artificial feature rules^14,17^, LSTM, CNN^24,25^, and BERT^6^. In paper 18, Lai et al. did not consider sentences with defective entities, but instead screened 9782 data out of 9870 for experiments, resulting in a relatively high average F1 score. The reason why papers 19 and 20 achieved such high results is that they did not consider the impact of ambiguity of entities with the same name, and only used fuzzy matching and other methods to find relevant entities in KB, while we fully considered the entity disambiguation task.

**Table 7:** NLPCC-ICCPOL 2016 KBQA results (%).

Model	Averaged F1
Xie et al.^14^	79.57
Yang et al.^25^	81.59
Xie et al.^24^	82.43
Lai et al.^17^	82.47
Liu et al.^6^	84.12
SUM(CoSENT)	85.94
Lai et al.^18^	86.60
Li et al.^19^	92.04
Lin et al.^20^	94.40

## Conclusion

We proposed a SUM to construct ODCKBQA. The proposed SUM fully considers the impact of ambiguity between entities with the same name, combines entity disambiguation and relation matching tasks within a unified framework, and uses a CoSENT model based on contrastive learning to learn deeper and more discriminative semantic vector representations. Through experimental results on the NLPCC ICCPOL 2016 KBQA datasets, prove the advantages of our proposed SUM model.

## Data Availability

The datasets generated and analyzed during the current study are available in the github repository, [https://github.com/haohuisss/SUM_A_Semantic_Union_Model_for_ODCKBQA].
